# Removal of the rate table: MEMS gyrocompass with virtual maytagging

**DOI:** 10.1038/s41378-023-00610-3

**Published:** 2023-11-06

**Authors:** Tongqiao Miao, Qingsong Li, Liangqian Chen, Junjian Li, Xiaoping Hu, Xuezhong Wu, Wenqi Wu, Dingbang Xiao

**Affiliations:** College of Intelligence Science and Technology, National University of Defense Technology, Changsha, 410073 China

**Keywords:** Electrical and electronic engineering, Engineering, Nanoscience and technology, Physics

## Abstract

High-performance micro-electro-mechanical system (MEMS) gyrocompasses for north-finding systems have been very popular for decades. In this paper, a MEMS north-finding system (NFS) based on virtual maytagging (VM) is presented for the first time. In stark contrast to previous schemes of MEMS-based NFSs (e.g., carouseling, maytagging) and the abandoning rate table, we developed a honeycomb disk resonator gyroscope (HDRG) and two commercial accelerometers for azimuth detection. Instead of the physical rotation of the integrated turntable in traditional NFSs, the vibratory working modes of the HDRG are rotated periodically with electronic control to reduce the uncertainty in the azimuth. After systematically analyzing the principle of NFSs with VM, we designed tests to verify the practicability at the sensor level. A bias instability of 0.0078°/h can be obtained during one day with VM in an HDRG. We also implemented comparative north-finding experiments to further check our strategy at the system level. The accuracy in the azimuth can reach 0.204° for 5 min at 28.2° latitude with VM and 0.172° with maytagging. The results show that without any mechanical turning parts, VM technology makes it possible to develop high-precision handheld MEMS NFSs.

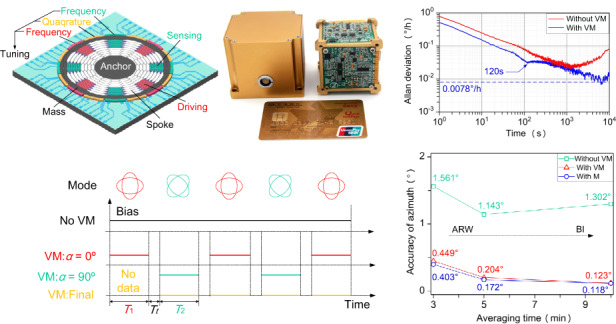

## Introduction

The orientation is indispensable for navigation and guidance, while north-finding with an accuracy of several milliradians (mrad) is now an urgent need for practical applications (e.g., targeting, pointing, dead reckoning, and inertial guidance)^1^. Therefore, the development of north-finding strategies has not been suspended in recent years. Digital magnetic compasses (DMCs) are widely used because of the advantages of low cost and integration. However, the working principle of DMCs (i.e., measuring the magnetic field of the Earth) indicates that the accuracy can be easily degraded in magnetically compromised environments^2,3^. GPS-based NFSs are another common orientation technology. With separated GPS antennas, the azimuth information of body can be easily obtained, but they fail in GPS-denied environments (e.g., indoor, jamming, underwater domain) because the satellite signals cannot be received^4,5^. The traditional method of using reference celestial bodies (e.g., the sun, Polaris) for orientation continues to this day. Although the error in measurement is controllable with excellent sensors and does not accumulate with time, it is seriously restricted by climatic conditions^6^.

Inertial NFSs, which are commonly called gyrocompasses, integrate inertial sensors (e.g., gyroscope and accelerometer) to find north. The orientation information can be obtained by measuring the Earth’s rotation and gravity vector. Without any external auxiliary information, the gyrocompass is not susceptible to interference and, therefore, provides an effective scheme for orientation under the complex and harsh environmental conditions mentioned above^7^. However, the Earth’s rotation rate is only approximately 15°/h, thus challenging the performance of gyroscopes^8^. Traditional devices such as dynamically tuned gyroscopes, ring laser gyroscopes and fiber optic gyroscopes have been generally used for accurate orientation, but they are bulky and expensive^9–13^. In recent years, great progress has been made in Coriolis vibratory gyroscopes, in which the long-term bias drift and environmental adaptability can be improved effectively in hemispherical resonator gyroscopes. However, the cost is still high for north-finding gyroscopes with an accuracy of several mrad^14,15^.

In contrast, with reduced size, weight, and power, MEMS gyroscopes have inherent advantages and can thus be implemented in the design of miniaturized and handheld NFSs^16^. Additionally, the continuous improvement in the performance makes it possible to achieve accuracies of several mrad in the azimuth with MEMS gyroscopes^17^. However, due to the limitations of the manufacturing process and control scheme, the long-term bias drift of MEMS gyroscopes still causes issues^18,19^. Making use of a continuous rotation to create a modulation of the Earth’s rotation rate, carouseling can be performed to identify the azimuth free from the influence of bias and scale-factor errors of the gyroscope^20^. Another technique that can be conducted to achieve bias rejection is maytagging, which is implemented by turning the gyroscope ±180°. Taking advantage of the gyroscope output in two positions, the bias can be observed and eliminated from the measured values of the azimuth^21^. However, both methods require a rate table, which restricts further integration and makes the system complicated.

In this paper, we established a MEMS gyrocompass with the VM technique. Without any physical rotation parts, we implemented an HDRG and two commercial accelerometers in a north-finding system. Instead of rotating the gyroscope with a rate table, the vibratory working modes of the HDRG are rotated periodically with electronic control to reduce the uncertainty of the azimuth. After systematically analyzing the principle of NFSs with VM, we designed tests to verify our scheme. The results show that the accuracy of the azimuth can reach 0.22° for a 5-min averaging time at 28.2° latitude with VM. Furthermore, the approach in this paper can also provide new ideas for other similar sensing and measurement systems^22,23^.

## Results

### Model of north-finding

**Fig. 1:**
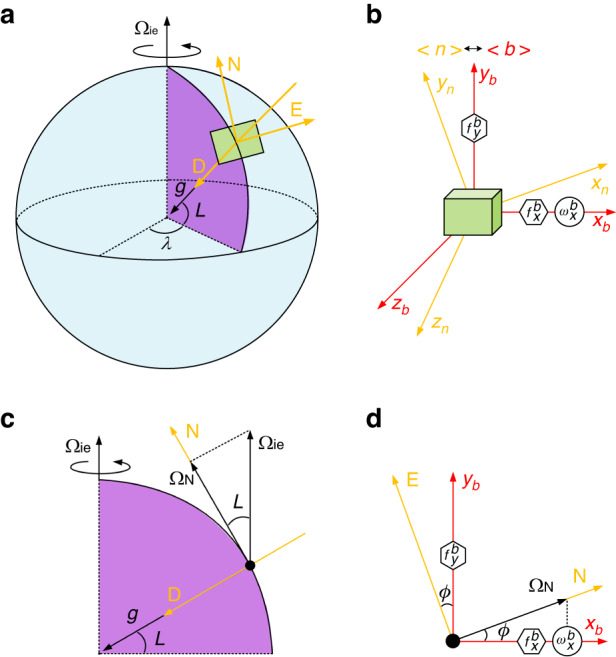
Schematic diagram of a north-finding system. **a** The Earth’s model and navigation reference frame (North-East-Down reference frame, i.e., < *n* > frame). **b** Rotation relationship between the < n > frame and body reference frame (*<* b > frame). **c** Cross-sectional view of the Earth’s model (North-Down plane, i.e., purple plane in Fig. 1a). **d** View of the local tangent plane (North-East plane, i.e., green plane in Fig. 1a)

As shown in Fig. 1a, the North-East-Down reference frame, which is commonly called the navigation reference frame ( < *n* > frame), is a local tangent plane reference system of the Earth’s surface and is used to calculate the orientation information of the body. Ω_*ie*_ (15.041067°/h) is the rotation rate of the Earth, *g* is the acceleration of gravity, and *λ* and *L* (28.2° N in Changsha, China) are the longitude and latitude, respectively. The *x*-axis, *y*-axis, and *z*-axis of the < *n* > frame (*x*_*n*_, *y*_*n*_, *z*_*n*_) point North, East, and Down. As shown in Fig. 1b, the body reference frame ( < *b* > frame) is rigidly attached to the body with a gyrocompass. The *x*-axis, *y*-axis, and *z*-axis of the < *b* > frame (*x*_*b*_, *y*_*b*_, *z*_*b*_) point Front, Right, and Below. An HDRG (*ω*^*b*^_*x*_) and two commercial accelerometers (${f}_{x}^{b}$, ${f}_{y}^{b}$) are installed on the < *b* > frame. According to the transformation of the Euler angle (i.e., heading angle *ϕ*, pitch angle *θ*, roll angle *γ*), the direction cosine matrix (DCM) from < *n* > frame to < *b* > frame (i.e., rotation relationship between two frames) can be expressed as:

1 $$\begin{array}{*{20}{l}}{{\bf{C}}_n^b = {R_x}(\gamma ){R_y}(\theta ){R_z}(\phi ) = \left[\begin{array}{*{20}{c}}1&0&0\\0&{\cos \gamma }&{\sin \gamma }\\0&{ - \sin \gamma }&{\cos \gamma }\end{array}\right]\left[\begin{array}{*{20}{c}}{\cos \theta }&0&{ -\sin \theta }\\0&1&0\\{\sin \theta }&0&{\cos \theta }\end{array}\right]\left[\begin{array}{*{20}{c}}{\cos \phi }&{\sin \phi }&0\\{ -\sin \phi }&{\cos \phi }&0\\0&0&1\end{array}\right]}\\{\qquad =\left[\begin{array}{*{20}{c}}{\cos \phi \cos \theta }&{\sin \phi \cos \theta }&{ - \sin \theta }\\{ - \sin \phi \cos \gamma + \cos \phi \sin \theta \sin \gamma }&{\cos \phi \cos \gamma + \sin \phi \sin \theta \sin \gamma }&{\cos \theta \sin \gamma }\\{\sin \phi \sin \gamma + \cos \phi \sin \theta \cos \gamma }&{ - \cos \phi \sin \gamma + \sin \phi \sin \theta \cos \gamma }&{\cos \theta \cos \gamma }\end{array}\right]}\end{array}$$

where *R*_*z*_(*ϕ*) represents the rotation around the *z*-axis at *ϕ* degrees, *R*_*y*_(*θ*) represents the rotation around the *y*-axis at *θ* degrees, and *R*_*x*_(*γ*) represents rotation around the *x*-axis at *γ* degrees. As shown in Fig. 1c, the Earth’s model can be obtained by analyzing the projection relationship in the < *n* > frame (e.g., *Ω*_*N*_ =*Ω*_*ie*_ cos*L*, is the projection of *Ω*_*ie*_ along North):

2 $$\begin{array}{l}{{\boldsymbol{\omega }}}_{ie}^{n}={[\begin{array}{l}{\varOmega }_{ie}\cos L \quad\, 0 \, -{\varOmega }_{ie}\sin L\end{array}]}^{T}\\ {{\bf{f}}}^{n}\,={[\begin{array}{l}0 \quad 0 \quad g\end{array}]}^{T}\end{array}$$

If we transform Eq. (2) to the < *b* > frame, the following can be obtained:

3 $$\begin{array}{l}{{\boldsymbol{\omega }}}^{b}={{\bf{C}}}_{n}^{b}{{\boldsymbol{\omega }}}_{ie}^{n}\\ {{\bf{f}}}^{b}\,=-{{\bf{C}}}_{n}^{b}{{\bf{f}}}^{n}\end{array}$$

Moreover, the ideal value of the sensors can be obtained by expanding Eq. (3) as follows:

4 $$\begin{array}{c}{\omega }_{x}^{b}={\varOmega }_{ie}\,\sin L\,\sin \theta +{\varOmega }_{ie}\,\cos L\,\cos \theta \,\cos \phi \\ {f}_{x}^{b}=g\,\sin \theta \\ {f}_{y}^{b}=-g\,\cos \theta \,\sin \gamma \end{array}$$

Actually, the heading angle *ϕ* can be calculated according to Eq. (4). More specifically, two accelerometers (${f}_{x}^{b}$, ${f}_{y}^{b}$) are used to measure the horizontal alignment (*θ* and *γ*) from the second and third expressions, while the HDRG (${\omega }_{x}^{b}$) is used to determine *ϕ* from the first expression. As shown in Fig. 1d, when *θ* and *γ* are zero, the relationship between ${\omega }_{x}^{b}$ and *ϕ* can be simplified in the North-East plane:

5 $${\omega }_{x}^{b}={\varOmega }_{ie}\,\cos L\,\cos \phi ={\varOmega }_{N}\,\cos \phi$$

### Operation of virtual maytagging

**Fig. 2:**
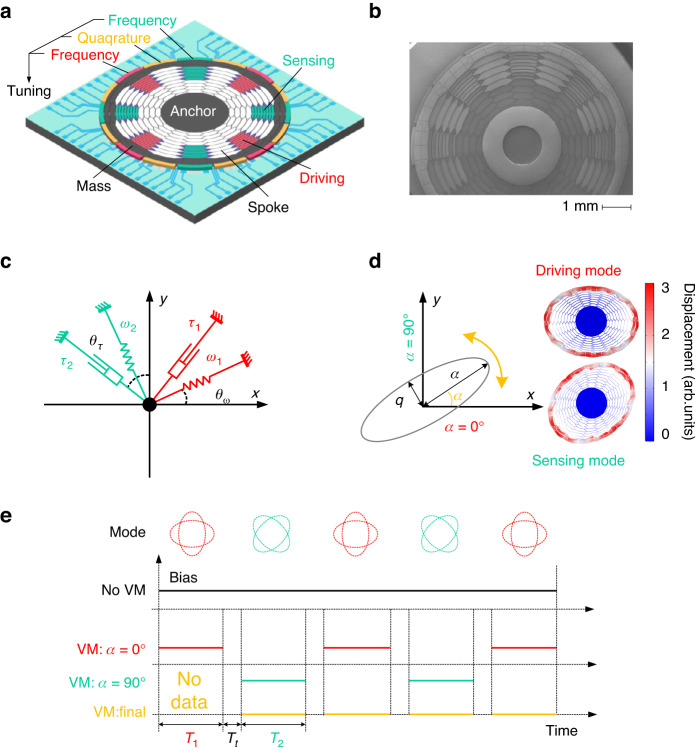
Operating principle of HDRG and VM. **a** 3D schematic diagram of the resonant structure in an HDRG. **b** SEM image of the resonant structure in the HDRG. **c** Equivalent two-dimensional harmonic oscillator model of an HDRG. Stiffness asymmetry and damping asymmetry of the resonator are considered in this model. **d** Vibration direction of the HDRG under the VM. The two subfigures on the right are the displacement fields of the driving and sensing modes (*n* *=* 2). The driving angles *α* *=* 0° and *α* *=* 90° form two working states of the VM. The driving mode is excited when *α* *=* 0°, and the sensing mode is excited when *α* *=* 90°. **e** Working sequence of the HDRG under VM. *T*_1_ and *T*_2_ are the durations of *α* *=* 0° and *α* *=* 90°, respectively. Moreover, both *T*_1_ and *T*_2_ are 120 s. *T*_t_ is the transition time of the two working states, which is 30 s

As shown in Fig. 2a, the resonator structure of the HDRG mainly consists of the central anchor, masses, spokes, and electrodes. The electrodes inside the resonator structure are used to measure and control the driving mode and sensing mode, while the electrodes outside are designed for quadrature tuning and frequency tuning. The SEM of the HDRG is shown in Fig. 2b. The diameter and thickness of the resonator structure are 8 mm and 148 μm, respectively. The resonator structure of an HDRG mainly consists of a central anchor, masses, spokes and electrodes. The HDRG is topologically reformed from the traditional nested-ring disk resonator. Instead of the concentric ring frame structure, the honeycomb-like frame structure is composed of hexagon-like units, which are interconnected with each other through shared spokes. The configuration of the lumped masses is designed based on the stiffness-mass decoupled principle to increase the quality factor (*Q*) of the HDRG. To increase the signal-to-noise ratio of the displacement detection, improve the voltage utilization efficiency, and complete the electrical adjustment of the resonator, electrodes are designed inside and outside the honeycomb structure^24^. Two silicon-on-insulator (SOI) wafers are used to fabricate the substrate and structure layer. First, the wire and anchor on the substrate are formed by deep reactive ion etching (DRIE). After generating the thermal oxide layer for protection, the substrate is bonded with a structure layer. Second, the aluminum pads are patterned for signal extraction after moving the handle wafer by chemical and mechanical polishing. Finally, the resonator structure and electrodes are formed by DRIE^25^.

As shown in Fig. 2c, d, the HDRG works on *n* = 2 wine-glass modes, which are composed of 0° and 45° modes with the same mode shape (i.e., driving and sensing modes, expressed *x*- and *y*-axes in rectangular coordinate system). The working frequency is 4.4 kHz, and the quality factor (*Q*) is 580k. The initial frequency split of the working modes is 0.2 Hz. Considering the two-axis lumped mass vibration model, the actual value of the HDRG that is applied in the force-to-rebalance (FTR) mode can be expressed as^26^:

6 $${\tilde{\omega }}_{x}^{b}={\omega }_{x}^{b}+b(\alpha )={\omega }_{x}^{b}+\varDelta (1/\tau )\sin 2({\theta }_{\tau }-\alpha )$$

where *b*(*α*) is the bias error of the HDRG and is the periodic function of the driving angle *α*. As shown in Fig. 2d, the vibration amplitude *a*, the quadrature motion *q* and the driving angle *α* are the orbital elliptic parameters that are used to describe the trajectory of the gyroscope. The angle that the ellipse makes with the *x*-axis is represented by *α*. *a* is the trajectory of vibration for a perfect gyroscope, while *q* is nonzero in the presence of imperfections (e.g., stiffness asymmetry, damping asymmetry). However, after quadrature tuning and frequency tuning in the HDRG, the trajectory is almost a straight line, that is, *a*>>*q*^25,27^. Δ(1/*τ*) represents the damping asymmetry of the resonant structure and can be calculated as Δ(1/*τ*) = 1/*τ*_1_ − 1/*τ*_2_. *τ*_1_ and *τ*_2_ are the attenuation coefficients of the damping principle-axis. *θ*_*τ*_ is the azimuth of damping. Actually, *b*(*α*) will drift with environmental factors (e.g., temperature, stress), therefore bringing uncertainty to the azimuth.

According to Eq. (6), it can be observed that the bias error of the HDRG *b*(*α*) has an opposite sign when *α* = 0° and *α* = 90°. At the same time, the ideal value of the HDRG remains unchanged, which provides the possibility for estimating and eliminating the bias error of the HDRG. Therefore, by substituting Eq. (5) into Eq. (6) and setting *α* equal to 0° and 90°, two equations can be obtained^28,29^:

7 $$\begin{array}{l}{{\tilde{\omega }}_{x}^{b}\Big|}_{\alpha ={0}^{^\circ }}={\omega }_{x}^{b}+\varDelta (1/\tau )\sin 2{\theta }_{\tau }={\varOmega }_{ie}\,\cos L\,\cos \phi +\varDelta (1/\tau )\sin 2{\theta }_{\tau }\\ {{\tilde{\omega }}_{x}^{b}\Big|}_{\alpha =9{0}^{^\circ }}={\omega }_{x}^{b}-\varDelta (1/\tau )\sin 2{\theta }_{\tau }={\varOmega }_{ie}\,\cos L\,\cos \phi -\varDelta (1/\tau )\sin 2{\theta }_{\tau }\end{array}$$

By summing the two equations in Eq. (7), the temperature drift of the HDRG can be eliminated, which is also the basic working principle of VM. In contrast to the bias rejection method of maytagging, which rotates the sensitive direction of the gyroscope *ϕ* to two discrete positions (0° and 180°) mechanically, virtual maytagging can compensate for the bias by electrical modulation without any physical turning parts. As shown in Fig. 2e, to obtain the measured value of the two working states in Eq. (7), the sequence diagram of the VM is designed for north-finding systems. *T*_1_ and *T*_2_ are the times of *α* = 0° and *α* = 90°, respectively, which are equal in length. There should be a transition time *T*_*t*_ between the two states for the rotation of *α*^27^. In this paper, the timing configuration is set as *T*_1_ = *T*_2_ = 120 s and *T*_*t*_ = 30 s for completing the north-finding process in 5 min. The ordinates of Fig. 2e depict the bias of the HDRG under different working states. When the HDRG operates in the traditional mode without VM, the bias will not be reversed (No VM). However, when the HDRG works under VM, the bias will be reversed periodically. Specifically, if the sign of bias is positive during *T*_1_ (VM: *α* = 0°), it will become negative during *T*_2_ (VM: *α* = 90°). It should be noted that the unstable working state of the HDRG causes the unavailability of bias during *T*_*t*_. Therefore, the bias of two adjacent work states can be added to estimate and eliminate the bias of the HDRG (VM: Final), which is also the reason for the lack of data during the first period of *T*_1_.

### Implementation of virtual maytagging

**Fig. 3:**
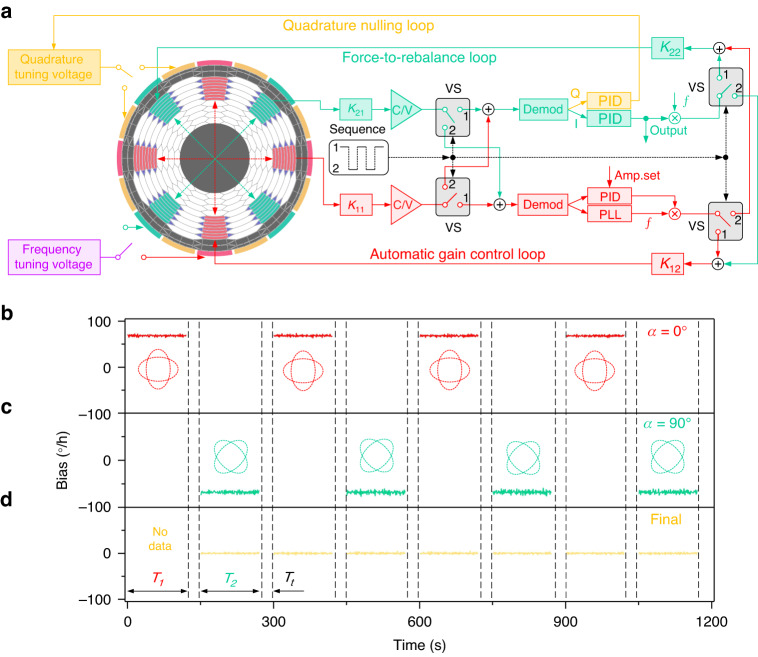
Implementation of VM and the experimental results of the working states. **a** Measurement and control scheme of the HDRG under VM. The virtual switch (VS) is used to change the working state of the HDRG. **b** Test results of the bias when the HDRG is working under *α* *=* 0°. **c** Test results of the bias when the HDRG is working under *α* *=* 90°. **d** Final bias of the HDRG according to the two working states (*α* *=* 0° and *α* *=* 90°) under VM. There is no final bias in the first working state when *α* *=* 0° because of missing data from the previous working state

As shown in Fig. 3a, there are three main measurement and control loops of VM in HDRG: the automatic gain control loop (AGCL), force-to rebalance loop (FTRL) and quadrature nulling loop (QNL). The AGCL is used for the exciting driving mode with constant amplitude *a*. First, the displacement signal of the driving mode passes through the C/V converter and becomes the voltage signal. Second, the amplitude and phase information of the vibration is obtained through the demodulation module. Finally, the PID controller and phase-locked loop are used to control the amplitude and phase, respectively. In both the FTRL and QNL, the vibration information of the sensing mode is used to control the HDRG. Regarding the FTRL, the in-phase signal (I) is used to suppress the displacement of the sensing mode, and the output of the HDRG (angular velocity) can be obtained by solving the rebalanced force. In the QNL, the quadrature-phase signal (Q) is utilized to eliminate the quadrature error of the HDRG, and the output of the PID controller in the QNL is the quadrature tuning voltage. In fact, to realize mode matching in an HDRG, a frequency tuning voltage is implemented. Approximately 5 mHz of circumferential frequency fluctuation can be observed^30^, which is within the mechanical bandwidth of the working mode (7.6 mHz).

The virtual switch (VS) is placed in the measurement and control loops of the HDRG. Two options (Option 1 and Option 2) are placed in the VS, which works according to the instructions of the sequence controller. Specifically, when Option 1 of the VS is selected, the driving mode is connected to the AGCL, and the sensing mode is connected to the FTRL. At this time, the HDRG works in the state of *α* = 0°. When Option 2 of the VS is selected, the driving mode and sensing mode are exchanged in the measurement and control loops. In this situation, the HDRG works in the state of *α* = 90°. Therefore, *α* can be controlled conveniently by setting the option of the VS. Since the VS and sequence controller can both be programmed in the FPGA, there is no need to add other auxiliary equipment to realize VM of the HDRG.

The adjustable gain module (*K*_11_, *K*_12_, *K*_21_, *K*_22_) is an indispensable part of the measurement and control loops of the HDRG. In contrast to the traditional working mode of the HDRG, under the working mode of VM, the measurement and control loops of the HDRG require periodic switching by the VS. As shown in Fig. 3a, if *K*_11_ ≠ *K*_21_ or *K*_12_ ≠ *K*_22_, the loop gains of the driving and sensing modes are not equal when the VS is set to Option 1 (*α* = 0°) and Option 2 (*α* = 90°). In these circumstances, the circuit noise, bandwidth, and range of the two working states are not the same, which affects the normal use of the HDRG under VM. Therefore, to match the above performance of the HDRG in two working states, the gain coefficient should meet the following relations:

8 $$\begin{array}{c}{K}_{11}={K}_{21}\\ {K}_{12}={K}_{22}\end{array}$$

The experimental results of bias in the HDRG are shown in Fig. 3b–d. According to Eq. (7), the sensitive direction of the gyroscope *ϕ* is set to 90° to eliminate the influence of *Ω*_*ie*_ on the bias measurement. When the HDRG works in the state of *α* = 0°, the mean bias is 68.15°/h. However, it is −67.85°/h in the working state of *α* = 90°. After the estimation and compensation based on the measured values of the two working states, the final mean bias of VM is 0.11°/h, which is reduced by more than two orders of magnitude. Therefore, it is proven that the VM technology can suppress the bias of the HDRG substantially.

### Design of the MEMS gyrocompass

**Fig. 4:**
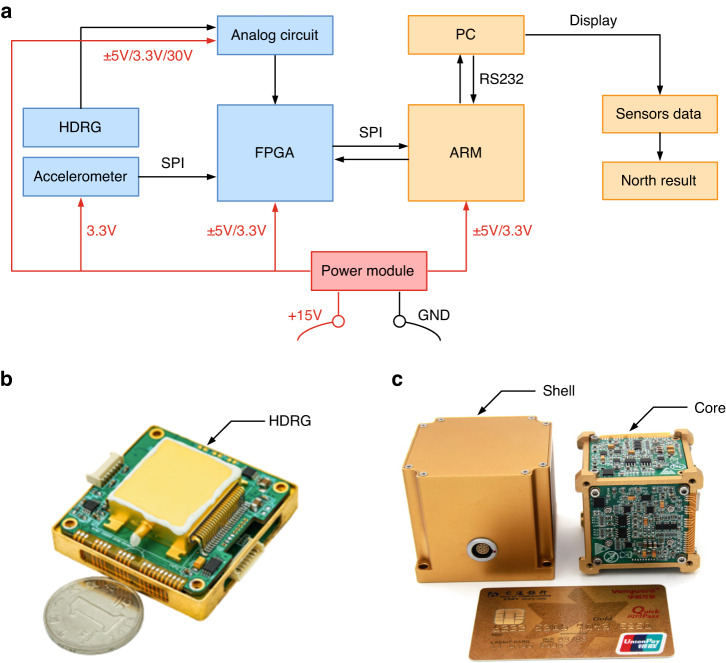
Schematic diagram of the hardware scheme and prototype of the MEMS gyrocompass. **a** Hardware scheme of the MEMS gyrocompass. It mainly includes the following three parts: the MEMS inertial sensors, north display, and power management module. **b** Photograph of a single HDRG. The size of a single HDRG is 60 mm × 50 mm × 20 mm. **c** Prototype of the integrated MEMS gyrocompass. The size is 60 mm × 60 mm × 60 mm, and the weight is 200 g

As shown in Fig. 4a, the hardware scheme of the MEMS gyrocompass includes the following three parts: the module of the MEMS inertial sensors, the north display and the power management system. The hardware circuit of the MEMS inertial sensors can realize the control and data reading of an HDRG and two accelerometers (ADXL357). Specifically, the control and output reading of the HDRG is realized by an analog circuit and the FPGA. While ADXL357 is one kind of digital MEMS accelerometer, the output can be read directly through the SPI without an additional hardware circuit.

To calculate and display the information of the north, the MCU is designed based on the embedded ARM microprocessor. The ARM receives the data of the MEMS inertial sensors from the FPGA and sends the control parameters of the HDRG to the FPGA through the SPI. Additionally, the north-finding algorithm is placed in the ARM, which can obtain the azimuth of the carrier through the data of the MEMS inertial sensors. The north-finding algorithm consists of two parts. In the first part, the measurement value of the HDRG is calibrated, and this value can be used to estimate and eliminate the bias of the HDRG. When the HDRG operates with VM, the corrected value *ω*^*b*^_*x*_ becomes half of the sum of the two expressions in Eq. (7) due to the rotation of the driving angle *α* to 0° and 90° electronically. In the second part of the north-finding algorithm, the azimuth of the carrier is calculated by the DCM. According to the second and third expressions in Eq. (4), the horizontal alignment (*θ* and *γ*) can be calculated through the measurement value of two accelerometers (${f}_{x}^{b}$, ${f}_{y}^{b}$). Additionally, the azimuth of Carrier *ϕ* can be obtained through the corrected value ${\omega }_{x}^{b}$ based on the first expression in Eq. (4). The communication mode between the PC and ARM is RS232. On the one hand, the external PC can display the output of the MEMS inertial sensors and north from the ARM. On the other hand, users can calibrate and debug the MEMS gyrocompass through the PC. The power management module provides the required voltage for each part of the hardware. The input of the module is +15 V, which can be supplied through the DC power supply or battery pack.

Before the integration and assembly of the MEMS gyrocompass, it is necessary to implement the preliminary performance evaluation and screening of the HDRG. As shown in Fig. 4b, the hardware circuit of the HDRG uses the modular design method, which can separate the analog circuit and FPGA. In this case, the HDRG can be easily assembled into the MEMS gyrocompass after selection by cable. The size of the single HDRG prototype is 60 mm × 50 mm × 20 mm. As shown in Fig. 4c, the overall size and weight of the developed MEMS gyrocompass prototype are 60 mm × 60 mm × 60 mm and 200 g, respectively.

### Sensor performance characterization

**Fig. 5:**
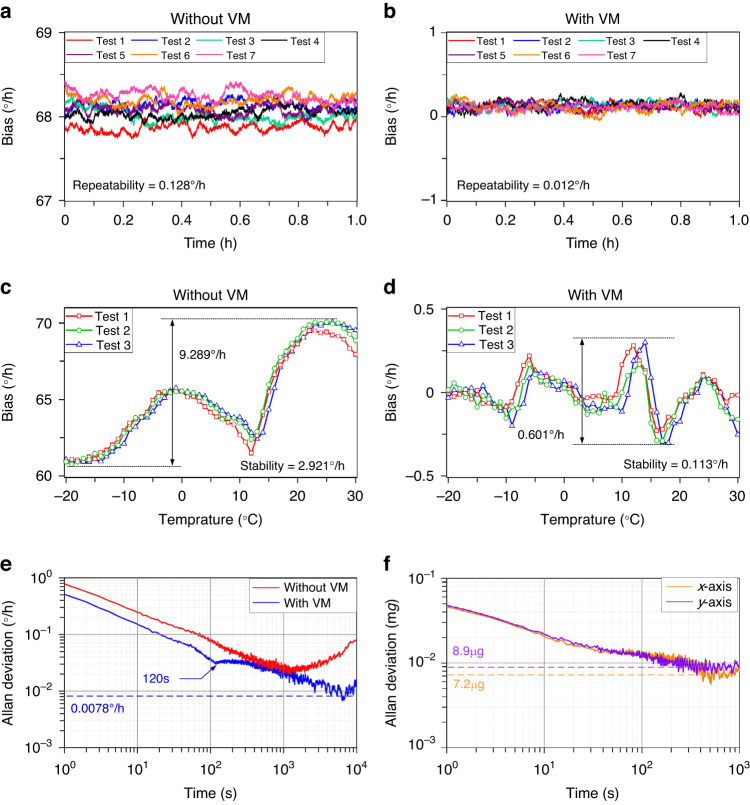
Performance characterization of the MEMS inertial sensors in a gyrocompass. **a** Bias repeatability of the HDRG without VM at room temperature. Seven tests of bias are conducted to evaluate the bias repeatability of the HDRG, and the sampling time is 1 s. **b** Bias repeatability of the HDRG with VM at room temperature. **c** Thermal bias repeatability of the HDRG without VM. The temperature range is from −20 °C to 30 °C. The test is conducted three times with a heating rate of 1 °C/min and a sampling time of 100 s. **d** Thermal bias repeatability of the HDRG with VM. **e** Allan deviation of the HDRG at room temperature. The calculated integration time reaches 10,000 s, and the original data are biased and continuously tested for 24 h with a sampling time of 1 s. **f** Allan deviation of the MEMS accelerometers along the x-axis and y-axis with a sampling time of 1 s at room temperature

The performance of the MEMS inertial sensors needs to be comprehensively analyzed and evaluated to verify the feasibility and effectiveness of VM. As shown in Fig. 5a, b, the bias repeatability of the HDRG is tested with and without VM. Seven tests are conducted in both cases at room temperature, and the power interruption for 1 h between every two tests is guaranteed. The sampling time of the original data is 1 s, and a 100 s moving average result of the raw data is shown for the convenience of observation and analysis. The results show that the bias repeatability is improved significantly from 0.128°/h to 0.012°/h with VM. Additionally, the thermal bias repeatability of the HDRG is tested. The temperature range is from −20 °C to 30 °C, and the heating rate is 1 °C/min. The test results of thermal bias repeatability (sampling time of 100 s) are shown in Fig. 5c and d. Without VM, the variation and stability of bias are 9.289°/h and 2.921°/h in the three tests. In comparison, the same indicators are improved to 0.601°/h and 0.113°/h with VM.

At the same time, typical Allan deviation for the HDRG and accelerometers at room temperature in static conditions is tested. The curve of the Allan deviation from the data of 24 h is shown in Figs. 5e and 5f. The bias instability of the HDRG is approximately 0.0078°/h at integration times of 7000 s with VM, which proves that the long-term bias drift of the HDRG is greatly suppressed. Significantly, the position of *α* is changed every 120 s between 0° and 90°, which will bring a repeatability error of bias and, therefore, cause the degradation of the Allan deviation curve in the middle integration time range^27^. Actually, this small degradation is also observed in traditional hemispherical resonator gyroscopes and is acceptable for practical applications^31^. The results also show the bias instability of the *x*-axis and *y*-axis accelerometers, which are 7.2 μg and 8.9 μg, respectively. Therefore, the orientation error of the MEMS gyrocompass caused by accelerometers can be ignored^32^.

### North-finding experiment

**Fig. 6:**
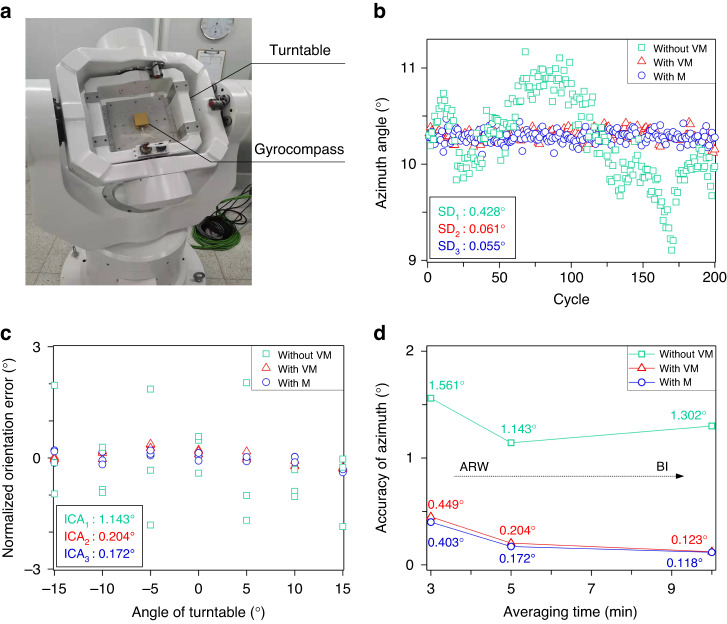
Results of the north-finding experiment. **a** Experimental setup of north-finding. The MEMS gyrocompass is fixed on the three-axis high-precision turntable. **b** North-finding results at a fixed position under different working modes: without VM, with VM, and with M. The experimental parameters: 0° (position of turntable), 5 min (time of north-finding), and 200 cycles (number of tests). **c** Internal coincidence accuracy of the north-finding results under different working modes. The experimental parameters were 0°, ±5°, ±10°, ±15° (positions of turntable), 5 min (time of north-finding), and 3 cycles (number of tests). **d** North-finding results with 3, 5, and 10 min under different working modes

The experimental setup of north-finding is shown in Fig. 6a, and a three-axis high-precision turntable is used as the azimuth reference of the MEMS gyrocompass. Three operating modes of MEMS gyrocompass are tested for comparison, which are seeking the north without virtual maytagging (without VM), with virtual maytagging (with VM) and with maytagging (with M). First, the north-finding experiment is carried out at the original position of the turntable, and the averaging time is set to 5 min. As shown in Fig. 6b, 200 cycles of each operating mode are conducted, and the standard deviation (SD) of the azimuth angle is 0.428° (without VM), 0.061° ( ≈ 1 mrad with VM) and 0.055° (with M). The results show that with VM and M, the SD is greatly reduced, and the SD is similar when the MEMS gyrocompass works under the VM and M modes.

The internal coincidence accuracy (ICA) of north-finding is tested for further verification. The azimuth angle of the turntable is rotated to seven positions (0°, ±5°, ±10°, ±15°), and the averaging time is set to 5 min. The results of three cycles are shown in Fig. 6c, and the ICA of north-finding is 1.143° (without VM), 0.204° (with VM) and 0.172° (with M). The improvement in the ICA is similar to that of the SD in Fig. 6b. Additionally, the ICA of north-finding at different averaging times (3, 5, and 10 min) is shown in Fig. 6d. With the extension of the alignment time, the ICA of north-finding is improved continuously with the working modes of VM and M, which means that the angle random walk of the HDRG becomes the leading source of orientation error in the MEMS gyrocompass. However, if the MEMS gyrocompass works in normal mode, the ICA will decline when the alignment time is 10 min. This is because the drift and nonrepeating property of bias destroys the bias instability (BI) of the HDRG, causing the BI to become the leading source of orientation error.

## Discussion

The performance of the reported MEMS gyrocompass is listed in Table 1. Through a comparison with other high-level MEMS-based NFSs, it can be found that the virtual maytagging MEMS gyrocompass presented in this paper exhibits excellent gyroscope BI and north-finding accuracy. Without any physical rotation elements, the method of virtual maytagging shows better potential in system reliability and integration. Future work will focus on the ASIC for the replacement of the PCB circuit board to further reduce the volume and weight of the MEMS gyrocompass.

**Table 1:** Performance summary of the MEMS gyrocompass

Work	BI of gyroscope	Accuracy	Method	Year
^1^	0.2°/h	5°@10 min	Carouseling	2013
^20^	0.1°/h	5°@10 min	Carouseling	2020
^21^	0.24°/h	1°@10 min	Maytagging	2021
^33^	0.027°/h	0.5°@3 min	Carouseling	2022
^34^	0.14°/h	0.5°@12 min	Carouseling	2019
^35^	0.02°/h	0.23°@4 min	Maytagging	2015
This work	0.0078°/h	0.20°@5 min	Virtual maytagging	2023

## Conclusion

In this paper, a MEMS NFS based on VM technology is presented for the first time. In contrast to the method of mechanical modulation with an integrated turntable, the electronic modulation of the HDRG is applied to the MEMS gyrocompass, which aims to suppress the long-term bias drift of the HDRG through the periodic rotation of vibration modes. Experiments are also carried out to verify the effectiveness of VM. The bias repeatability of the HDRG is improved from 0.128°/h to 0.012°/h, and the bias instability reaches 0.0078°/h at integration times of 7000 s. Furthermore, the SD of north-finding at a fixed position is 0.061° (≈1 mrad), and the ICA of seven positions can reach 0.204° at an averaging time of 5 min with VM, which is similar to that of mechanical modulation (i.e., 0.055° and 0.172°). Therefore, high-precision MEMS NFSs can potentially be developed without any mechanical turning parts.
